# Meal for Two: Human Cytomegalovirus-Induced Activation of Cellular Metabolism

**DOI:** 10.3390/v11030273

**Published:** 2019-03-19

**Authors:** Irene Rodríguez-Sánchez, Joshua Munger

**Affiliations:** 1 Department of Microbiology and Immunology, School of Medicine and Dentistry, University of Rochester, Rochester, NY 14642, USA; Irene_RodriguezSanchez@URMC.Rochester.edu; 2 Department of Biochemistry and Biophysics, School of Medicine and Dentistry, University of Rochester, Rochester, NY 14642, USA

**Keywords:** human cytomegalovirus, HCMV, metabolism, host–virus interactions, glycolysis, glutaminolysis, amino acid, fatty acids, pyrimidine, mTOR

## Abstract

Viruses are parasites that depend on the host cell’s metabolic resources to provide the energy and molecular building blocks necessary for the production of viral progeny. It has become increasingly clear that viruses extensively modulate the cellular metabolic network to support productive infection. Here, we review the numerous ways through which human cytomegalovirus (HCMV) modulates cellular metabolism, highlighting known mechanisms of HCMV-mediated metabolic manipulation and identifying key outstanding questions that remain to be addressed.

## 1. Introduction

Human cytomegalovirus (HCMV) is an opportunistic pathogen that causes substantial morbidity in immunosuppressed populations, including transplant recipients, cancer patients undergoing immunosuppressive therapies, and AIDS patients [1,2]. HCMV infection is also a major cause of allograft rejection in a wide variety of different tissue transplant recipients [3]. Further, HCMV causes significant congenital morbidity with central nervous system damage occurring in the majority of symptomatic newborns [2,4]. While anti-HCMV therapeutics exist, they suffer from poor bioavailability [5], off-target toxicity [6], and the emergence of drug resistant viral strains [7,8]. In the absence of a protective vaccine, the development of novel effective anti-HCMV therapeutics would substantially curtail HCMV-associated morbidity.

Over the past decade, the number of manuscripts describing how viruses modulate host-cell metabolism has greatly expanded. It is now clear that many viruses have evolved to target specific metabolic enzymes and the mechanisms of their regulation and, further, that these interactions with the metabolic network are critical for successful infection (reviewed in [9,10]). However, the field is at the very earliest stages of elucidating the mechanisms through which viruses modulate specific metabolic activities, and determining how they contribute to successful infection. Addressing these issues will further our understanding of an essential host–pathogen interaction, with the potential to identify novel targets for therapeutic intervention. Below, we review what is known about how HCMV interacts with the cellular metabolic network, and raise key areas requiring further investigation. 

## 2. HCMV-Mediated Modulation of Glycolytic Metabolism

**Figure 1:**
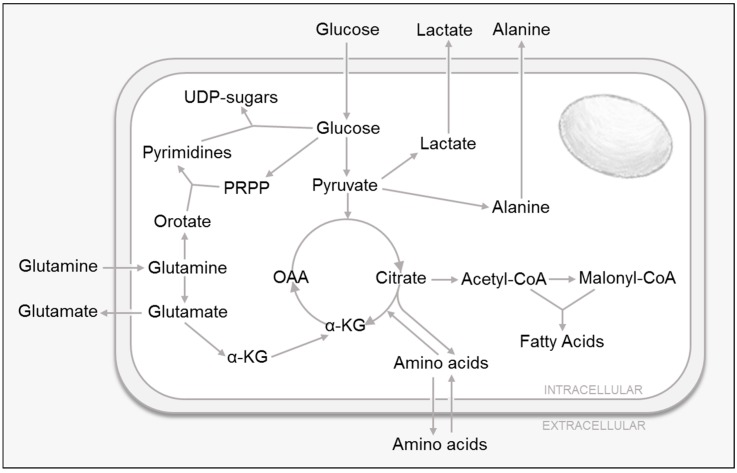
Central Carbon Metabolism.

Glucose is an essential nutrient that drives cellular energy production, while also functioning as a critical resource that provides molecular building blocks via various catabolic pathways such as glycolysis, the tricarboxylic acid (TCA) cycle and the pentose phosphate pathway [11] (Figure 1). Over thirty years ago, one of the first studies that linked HCMV to cellular metabolic modulation reported that glucose uptake was significantly increased in HCMV-infected cells, and that this metabolic induction required active viral replication [12]. More recently, studies have shown that HCMV infection not only induces glucose consumption [13,14,15,16], but it also increases glucose transport [13,16], glycolytic pool sizes [14,17] and glycolytic effluxes [14,17,18].

In line with the studies demonstrating that HCMV infection induces glucose uptake [13,14,15,16], HCMV infection was shown to dramatically increase the expression of the adipose tissue-specific glucose transporter type 4 (GLUT4) [16,19], and eliminate the expression of the ubiquitously distributed GLUT1 in infected fibroblasts [16]. This switch to a non-predominant higher-capacity glucose transporter seems to be essential for the success of viral infection, as the pharmacological inhibition of GLUT4 lowers virally-induced glucose uptake and greatly impacts virus production [16]. The mechanism responsible for this induction is still unclear, but it appears to require early viral protein synthesis and expression of the HCMV immediate early protein (IE) IE72 [16]. In addition, the induction of GLUT4 levels appears dependent on the HCMV-mediated activation of AMP-activated protein kinase (AMPK), a major energy regulating kinase [19].

Consistent with the observed increases in glucose uptake, LC–MS/MS-based metabolic flux analysis has shown that infected cells exhibit increased glycolytic fluxes as shown by the rapid conversion of heavy labeled glucose into labeled glycolytic intermediates [13,18]. In addition, HCMV-infected cells show an increased rate of lactate excretion [17,18], as well as an increase in the abundance of several intracellular glycolytic intermediates such as fructose 1, 6-bisphosphate (FBP), dihydroxyacetone phosphate (DHAP), 3-phosphogylcerate (3PG), and phosphoenolpyruvate (PEP) [17,18]. This induction of glycolytic flux is consistent with previous gene expression studies showing that HCMV infection upregulates several metabolic and biosynthetic enzymes that regulate glycolysis, including phosphofructokinase (PFK), pyruvate dehydrogenase (PDH), pyruvate kinase (PK), AMP-activated protein kinase (AMPK) and Ca^2+/^calmodulin-dependent protein kinase kinase (CaMKK) [13,14,19]. 

Glycolysis is important for viral infection, as its inhibition attenuates HCMV DNA replication [13] and virus production [20]. However, the exact mechanisms responsible for HCMV-mediated glycolytic induction are still not completely understood. They appear to require viral gene expression but not DNA replication [13]. Interestingly, previous work has shown that the inhibition of either AMPK or an AMPK activating enzyme, i.e., CaMKK, blocks virally-induced glucose uptake [19] and glycolytic flux [13,19], as well as HCMV replication [13,19]. While suggestive, the functional linkages between CaMKK and AMPK, glycolytic activation and productive viral replication remain unclear. Importantly, the targeting of CaMKK or AMPK has little effect on mock-infected glycolytic activity or cell survival, but greatly inhibits virally-induced glycolytic activation and viral replication [13,19], thus highlighting the possibility of targeting CaMKK or AMPK for therapeutic development. 

More recently, the HCMV immediate early U_L_38 protein was implicated in glycolytic activation [17]. It was shown that U_L_38 expression is both necessary and sufficient to induce both glucose consumption and lactate secretion [17]. Previously, the U_L_38 protein was reported to bind and antagonize the tuberous sclerosis protein complex 2 (TSC2), a major suppressor of the mammalian target of rapamycin (mTOR). The U_L_38-mediated inhibition of TSC2 thus allows for mTOR activation under inhibitory conditions [21,22]. Previous work has demonstrated that HCMV infection activates mTORC1 and that maintenance of this activity is required for high-titer viral replication [23,24]. mTOR is a serine/threonine kinase that coordinates cell growth, proliferation and metabolism by controlling the balance between anabolic and catabolic processes in response to environmental cues, such as nutrients or growth factors [25,26,27]. Although U_L_38 was reported to induce glucose consumption and glycolysis [17], as well as induce mTOR activation [21], it appears that U_L_38’s activation of glucose metabolism is independent of mTOR activation. Notably, U_L_38’s glycolytic activation is dependent on its inhibition of TSC2, suggesting that TSC2 has important functions that are mTOR independent [17]. 

It is clear that HCMV strongly activates glucose metabolism in infected cells, and that glycolysis is important for productive infection. Less clear are the mechanisms through which glycolysis contributes to HCMV infection. The most obvious contribution to infection would be the production of energy in the form of ATP [20] or NADH [14], which could support the energetically expensive biosynthetic reactions required to generate massive amounts of viral proteins, nucleic acids, and envelope. This idea is supported by previous work which suggests that HCMV infection greatly affects mitochondria biogenesis and their function. HCMV infection was found to induce mitochondrial DNA (mtDNA) accumulation [28], mitochondrial transcription and translation systems [29], mitochondrial-encoded respiratory chain components [29] and the expression of oxidative-phosphorylation genes [30]. Interestingly, knock-down of the mitochondrial RNA methyltransferase 3 (MRM3), a mitochondrial-encoded respiratory chain component, was found to inhibit viral growth under bioenergetically restricting conditions [29]. In addition to its function in energy production, glucose and its catabolic byproducts serve as the molecular building blocks for a wide variety of macromolecules produced during infection, including fatty acids, phospholipid head groups, and nucleic acids. Further, glucose-derived carbon provides the main pool of a number of post-translation modifications that are critical for successful infection. For example, glycolytic activation provides the glycosyl groups that drive increased pyrimidine biosynthesis and UDP-sugar biosynthesis that were found to be required for viral envelope protein glycosylation [31].

## 3. HCMV and Cellular Amino Acid Metabolism

In addition to being the monomeric units required for protein synthesis, amino acids are also capable of supporting cellular energy supplies via feeding the TCA cycle [11] (Figure 1). Further, amino acid catabolism also provides metabolic building blocks to support other metabolic pathways, e.g., nitrogenous base and amino sugar synthesis. Of particular importance to these pathways is glutamine, the most abundant amino acid in the body. Glutamine can be used as a carbon source to replenish the TCA cycle, produce glutathione, the major non-enzymatic cellular anti-oxidant, and serve as a precursor to nucleotides, nonessential amino acids and lipids [11]. Glutaminolysis is the process by which cells convert glutamine into α-ketoglutarate to feed the TCA cycle. Glutamine is first converted into glutamate, which is further metabolized into α-ketoglutarate via two different pathways. The first step involves glutamate dehydrogenase, which generates the byproduct ammonium and NADH, which can be later oxidized via respiration. The second utilizes transaminases, which allow for the generation of nonessential amino acids such as aspartate, alanine and P-serine. In addition, α-ketoglutarate can also be metabolized into acetyl-CoA for fatty acid biosynthesis or aspartate for nucleotide synthesis (reviewed in [32,33]). 

The first studies linking HCMV and glutamine metabolism found that HCMV infection induces glutamine uptake [17,18,20], as well as glutaminolysis, as shown by an increased secretion of glutamate [17,18] and ammonia [20]. In line with these data, it has also been shown that HCMV infection induces the activity of several enzymes involved in glutaminolysis such as glutaminase (GLS) and glutamate dehydrogenase (GDH) [20]. Further, despite not being an essential amino acid, glutamine utilization is required for productive HCMV infection [20]. Glutamine deprivation significantly lowers ATP levels in infected cells while having little effect on mock-infected cells [20]. 

The mechanisms through which HCMV activates glutamine metabolism are still largely unclear. However, it was recently found that U_L_38 is necessary and sufficient to induce glutamine consumption and glutamate secretion [17]. As mentioned above, the U_L_38 protein was reported to bind and antagonize TSC2 to allow mTOR activation [21,22], which is required for high-titer viral replication [23,24]. Similarly to what was observed for glucose metabolism, U_L_38’s activation of glutamine metabolism is independent of mTOR activation but dependent on its inhibition of TSC2 [17]. Furthermore, TSC2 knock-down phenocopies the majority of phenotypes associated with U_L_38 expression [17]. The mTOR-independent mechanisms through which the U_L_38–TSC2 interaction impacts glutamine metabolism remain to be elucidated. 

While it is not completely clear how glutamine contributes to HCMV infection, clues have begun to emerge. HCMV infection activates the TCA cycle [18], the various pools of which can largely be maintained by glutamine catabolism [20]. Glutamine tracer studies have suggested that the α-ketoglutarate derived from glutaminolysis is a major supplier of TCA cycle pools and that less than 30% of the produced malate pools come from glucose [18]. The link between TCA cycle pools, and glutamine catabolism is functionally important as supplementation with α-ketoglutarate can rescue ATP and viral production observed under glutamine deprivation [20]. These data suggest that in HCMV-infected cells, glutamine is important to maintain TCA cycle pools.

Despite the essential role other amino acids play in central carbon metabolism, more work is required to fully understand how HCMV infection affects cellular amino acid dynamics. Early work showed that HCMV infection induces intracellular alanine levels [14]. Later work revealed that HCMV induces substantial changes to cellular amino acid metabolism. HCMV infection induces the consumption of leucine/isoleucine, arginine, serine and valine among others, as well as the secretion of proline, ornithine and alanine [17]. These results suggest that HCMV reprograms cellular amino acid metabolism; however, the biological consequences of these activities on viral replication are still largely unknown. 

The mechanisms responsible for HCMV-induced amino acid reprogramming are not completely clear. It was shown that expression of the U_L_38 protein is both necessary and sufficient to induce the previously mentioned amino acid fluxes [17]. Similarly to what was observed for glucose and glutamine metabolism, it seems that U_L_38’s activation of several amino acid fluxes such as alanine or proline is actually independent of mTOR activation, but dependent on its inhibition of TSC2 [17]. However, in some cases, such as arginine consumption, the HCMV-induced amino acid changes are sensitive to mTOR inhibition [17]. Collectively, these data reflect the multifaceted regulation of amino acid metabolism during infection.

Aside from glutamine, the functional consequences of the HCMV-mediated changes to amino acid metabolism remains to be assessed. The simplest possibility would be that induction of amino acid consumption could field the increased protein translation observed during different stages of infection [34]. Alternatively, amino acids can provide a wide range of other metabolic intermediates such as pyruvate, α-ketoglutarate, succinyl-CoA, fumarate, oxaloacetate, acetyl-CoA or acetoacetate that can have diverse cellular impacts [11]. The secretion of alanine, for example, can enable glutamine-driven TCA cycle anaplerosis. This process requires the transfer of nitrogen from glutamate to pyruvate, the acceptor molecule, to form α-ketoglutarate and alanine, and can help dispose of waste ammonia. The induction of such activities was found to be critical in cancer cells [35]. Additionally, the increase in leucine/isoleucine consumption can potentially supply the acetyl-CoA [11] required for enhanced fatty acid biosynthesis and necessary for envelope biogenesis. Amino acids not only provide intermediates for metabolic processes, but they are also capable of driving protein kinase regulatory networks [36]. For example, leucine and arginine can activate mTORC1 [21,22,36], which as mentioned above, was shown to be required for high-titer viral replication [23,24]. This suggests the possibility that increasing the consumption of specific amino acids could actually contribute to mTOR activation during infection. 

## 4. HCMV Induces Pyrimidine and UDP-Sugar Biosynthesis

**Figure 2:**
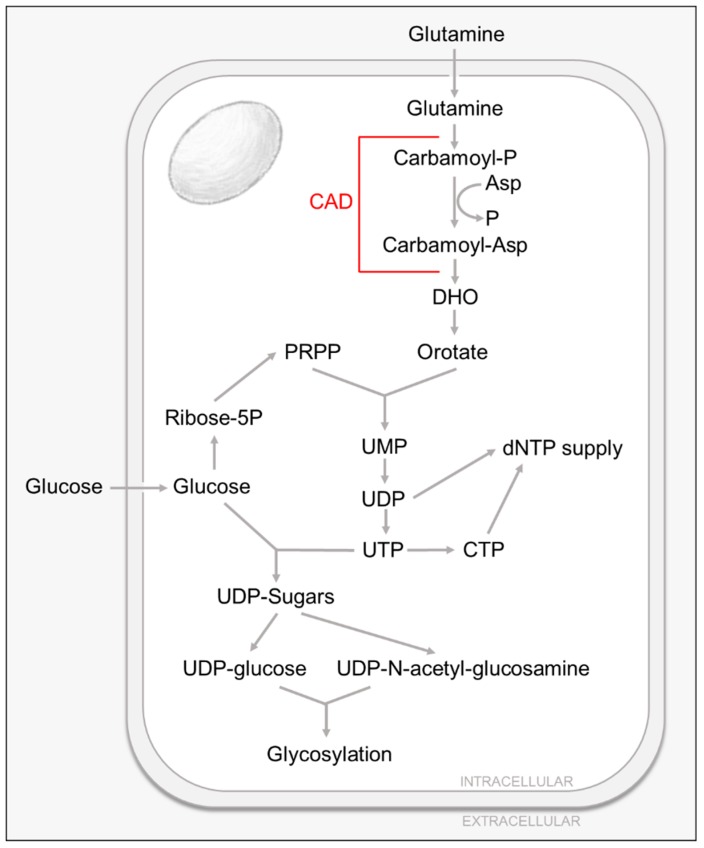
Pyrimidine and Nucleotide Sugar Metabolism.

Pyrimidine nucleotides play a critical role in cellular metabolism, serving as precursors of RNA, DNA and cytidine diphosphate-diacylglycerol (CDP-DAG), which serves as a basic component of cellular membranes, an intermediate lipid metabolite, as well as a key element in lipid-mediated signaling [37,38,39]. In addition, pyrimidine nucleotides, specifically UTP, are essential for the production of UDP-sugars used in protein glycosylation and glycogen synthesis [37,38,39]. UDP-glucose, UDP-N-acetyl-glucosamine (UDP-GlcNAc) as well as GDP-mannose are required for N-linked glycosylation, whereas O-linked glycosylation requires UDP-N-acetyl-glucosamine [37,38,39] (Figure 2).

Pyrimidines can be generated via two different routes. Nucleotides can be recycled by the salvage pathways or synthesized de novo from metabolic intermediates. Most cells have several specialized passive and active transporters [40] that allow for the reutilization and conversion of extracellular pyrimidine nucleosides into nucleotides by the salvage pathways (reviewed in [37]). As for the de novo pathway, the ring structure is assembled through a multistep pathway that utilizes glutamine and aspartate and employs the function of the rate-limiting, multistep-enzyme carbamoyl phosphate synthetase-aspartate transcarbamylase-dihydroorotase (CAD). CAD’s product, dihydroorotate (DHO), combined with phosphoribosyl pyrophosphate (PRPP), is ultimately converted to the nucleotide UMP (reviewed in [37]). In general, the de novo pyrimidine biosynthesis pathway is minimally active in resting or fully differentiated cells where the need for pyrimidines is largely satisfied by the salvage pathways. The de novo pathway, however, is indispensable in proliferating cells in order to satisfy the increased demand for nucleic acids and other cellular components [37,41].

The first study highlighting the importance of pyrimidine biosynthesis for HCMV infection demonstrated that the pyrimidine biosynthesis inhibitor, leflunomide, attenuates viral production of several clinical isolates in both fibroblasts and endothelial cells, and that it appears to interfere with virion assembly [42]. In addition, this agent reduced viral load in vivo [42]. Later studies showed how HCMV infection increases intracellular pyrimidine metabolite pools without significantly affecting purine pool levels [14,31], and that this induction can be blocked by the use of pyrimidine biosynthesis inhibitors [31]. Notably, these changes in metabolic pools are HCMV specific, as the extent of concentration changes exceeds those found in the switch between the quiescent and growing states of uninfected fibroblasts [14]. Metabolic flux analysis using liquid chromatography–tandem mass spectrometry and heavy labeled glucose show that HCMV infection also induces pyrimidine biosynthesis [31].

HCMV-induced pyrimidine biosynthesis appears essential for successful viral infection. The pharmacological inhibition of pyrimidine biosynthesis attenuates the production of viral progeny and this inhibition can be rescued by the addition of exogenous uridine [31]. The mechanism behind this induction is still not completely understood, although it seems to be dependent on viral gene expression, but not viral DNA replication [31]. HCMV infection induces the activating phosphorylation of the rate-limiting enzyme of pyrimidine biosynthesis, CAD. Further, CAD appears to be important for infection, as treatment with CAD-specific siRNA attenuates the production of infectious viral progeny [31].

HCMV-induced activation of pyrimidine biosynthesis can not only provide nucleotides for viral genomes and RNA, but it is also essential for the production of UDP-sugars used in protein glycosylation. As mentioned previously, UTP is required for the formation of UDP-glucose and UDP-GlcNAc, which are the essential subunits that drive glycosylation reactions. Previous work has shown that the HCMV viral envelope contains a number of glycoproteins that are critical for HCMV replication, with functions that range from tegument assembly, envelope formation and host cell entry [43,44].

The first studies highlighting the importance of glycosylation reactions for HCMV infection showed that the inhibition of UDP-GlcNAc transferases by the pharmacological inhibitor tunicamycin inhibits HCMV replication [45]. HCMV infection not only increases intracellular UDP-sugar metabolite pools, UDP-glucose and UDP-GlcNAc among them [31], but it also induces UDP-sugar biosynthesis as shown by an increase in both UDP-glucose and UDP-GlcNAc biosynthetic flux [31]. Further, the induction of both UDP-sugar metabolic pools and biosynthetic fluxes during HCMV infection were dependent on de novo pyrimidine biosynthesis, as they were largely inhibited by the use of a pharmacological inhibitor against CAD [31]. The dependence of UDP-sugar metabolism on de novo pyrimidine biosynthesis was not observed during mock-infection, suggesting that the induction is HCMV specific [31]. In addition, further studies suggest that HCMV relies on increased pyrimidine biosynthesis to provide the UDP-sugars necessary for protein glycosylation. The pharmacological inhibition of the rate limiting pyrimidine biosynthetic enzyme CAD resulted in decreased abundance of the glycosylated form of the essential HCMV glycoprotein gB, as well as an increased accumulation of the unglycosylated form [31]. In addition, this inhibition was rescued with UDP-sugar or uridine supplementation, which suggests that glycosylation of gB is highly dependent on de novo pyrimidine biosynthesis [31]. Notably, the glycosylation state of a host cell glycoprotein CD44S was not impacted by CAD inhibition [31], suggesting the possibility that de novo UDP-sugar biosynthesis could specifically be funneled towards the glycosylation of viral proteins. 

Although the mechanisms are unknown, it is clear that the induction of UDP-sugar metabolism during HCMV infection seems to be essential for successful viral infection, as the inhibition of UDP-GlcNAc transferases by the pharmacological inhibitor tunicamycin inhibits HCMV replication [45] and the production of infectious virions [31]. Further, the use of pyrimidine biosynthesis inhibitors PALA or A3, which target CAD and dihydroorotate dehydrogenase (DHODH) respectively, inhibit HCMV replication [31]. Interestingly, this inhibition could be rescued with supplementation with uridine, UDP-GlcNAc or orotate [31]. These data suggest that HCMV infection requires the induction of pyrimidine biosynthesis to not only generate new viral genomes and RNA, but to produce the UDP-sugar pools required for viral protein glycosylation.

## 5. HCMV Infection Induces Fatty Acid Biosynthesis

**Figure 3:**
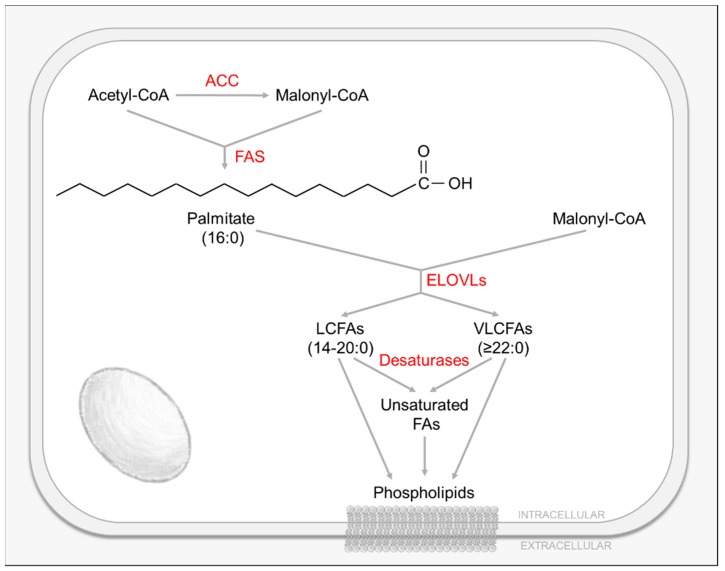
Fatty Acid Biosynthesis, Elongation and Desaturation.

Lipids, and their constituent fatty acids (FAs), play a wide variety of critical roles in mammalian cells (Figure 3). Most simply, they are constituents of cellular membranes that segregate organisms from their environment, and are a key physical component in the maintenance of diverse biochemical gradients. In addition, FAs, as post-translational modifications, are critical to the function of many proteins, and also serve as a means to store energy and carbon, e.g., triacylglycerides. Lipids and FAs also act as potent signaling molecules, e.g., diacylglycerol (DAG) and phosphatidylinositol-3,4,5-trisphosphate (PIP3), and serve as the building blocks for many primary and secondary metabolites including vitamins, steroid hormones and prostaglandins [11]. These FAs are derived either from dietary sources or are synthesized de novo by the cell. Most human tissues preferentially use dietary (exogenous) lipids for synthesis of new structural lipids, while de novo (endogenous) FA synthesis is usually suppressed [11,46].

De novo FA synthesis occurs in the cytoplasm and requires acetyl-CoA, mainly generated from citrate [11]. Cytoplasmic citrate is produced either by the TCA cycle and shuttled from the mitochondrion to the cytosol, or from cytoplasmic α-ketoglutarate derived from the reductive carboxylation of glutamine by isocitrate dehydrogenase-1 (IDH1) [11,46]. Citrate is then converted to acetyl-CoA and oxaloacetate (OAA) via ATP-citrate lyase (ACL) [11,46]. Cytoplasmic acetyl-CoA is then transformed to malonyl-CoA by the FA biosynthesis rate-limiting enzyme acetyl-CoA carboxylase (ACC) [11,46] (Figure 3). During FA biosynthesis, the serial condensation of seven malonyl-CoA molecules and one priming acetyl-CoA by the multifunctional fatty acid synthase (FAS) enzyme produces palmitate (16 carbons) [11,46]. Palmitate can be further processed to make long chain FAs (LCFAs, 14–20 carbons) and very long chain FAs (VLCFAs, ≥22 carbons), or processed to include desaturations by introducing double bonds among its carbons [11,46]. In human cells, FA elongation is carried out by one or more of seven FA elongases (ELOVL1-7) by adding two carbon units from malonyl-CoA to the pre-existing FAs [47] (Figure 3). These resulting FAs can be used to generate triglycerides, phospholipids or energy [47,48]. Similarly to FA biosynthesis, sterol biosynthesis requires acetyl-CoA as a precursor and is controlled by the rate limiting enzyme 3-hydroxy-3-methylglutaryl coenzyme A (HMG-CoA) reductase. The resulting isoprenoid groups can be incorporated into many end-products including cholesterol, ubiquinone, heme, dolichol, etc [11].

Lipid homeostasis is essential for the cell, as they are the main determinants of membrane permeability, fluidity, organelle identity and protein function [49]. Regulation of the cellular lipid environment is achieved via both transcriptional and post-transcriptional mechanisms. Sterol regulatory element binding proteins (SREBPs) and their corresponding sterol response elements (SREs) are crucial transcriptional regulators of many lipogenic genes, such as acetyl-CoA carboxylase (ACC), fatty acid synthase (FAS) or ELOVLs [50,51]. SREBPs are a family of transcription factors that are synthetized as inactive precursors anchored to the endoplasmic reticulum (ER) membrane. They form a complex with their chaperone SREBP cleavage activation protein (SCAP) [50]. Under low sterol levels, SREBPs are proteolytically cleaved and activated [50]. Activated SREBPs translocate to the nucleus, where they bind SREs found in the promoters of many cholesterol and fatty acid synthetic genes, thus upregulating their expression [50]. Several SREBPs were described to regulate different aspects of cellular lipid homeostasis, with SREBP1 controlling the expression genes involved in both fatty acid and cholesterol biosynthesis, and SREBP2 preferentially controlling genes involved in cholesterol homeostasis [52].

The first study to examine the effects of HCMV infection on cellular lipid homeostasis found that infection activated the citrate shuttle, and that the citrate, malonyl-CoA and acetyl-CoA levels were increased [14,18]. Later studies showed that HCMV infection upregulated de novo FA biosynthesis [18,53,54], as well as induced the activity and expression of the rate-limiting FA biosynthetic enzyme ACC1 [53,54]. This virally-induced activation of ACC1 is essential for the observed increase in FA biosynthesis, as ACC1 inhibition by RNAi greatly inhibited virally-induced FA biosynthesis [53]. HCMV infection was also shown to induce FA elongation [55,56] and the accumulation of VLCFAs (≥26 carbons) [55,56]. Most likely these VLCFAs are incorporated into the viral envelope, which was reported to be enriched in saturated VLCFAs [55,56]. It was found that the expression of several FA metabolic enzymes involved in the production of VLCFAs is induced during infection [55,56]. ELOVL7, specifically, was shown to be required for HCMV-induced FA elongation, as its knock-down greatly inhibits the production of VLCFAs during infection [55]. Several studies have shown that HCMV infection also induces the activation of lipogenic transcription factors, SREBP1 [54] and SREBP2 [53], possibly by a SCAP-dependent process [54]. HCMV-induced SREBP activation appears to be independent of the typical regulation by cellular sterol levels [54]. Further, SREBP activation is required for the HCMV-mediated induction of FA biosynthesis [53,54,55], as well as the enhanced expression of lipogenic genes observed during infection, such as ACC1 and ELOVL7 [53,54,55]. Interestingly, HCMV infection has also been found to alter sterol metabolism. HCMV infection of lung fibroblasts and smooth muscle cells was found to increase cellular levels of neutral lipids, such as cholesterol [57]. In addition, it was shown that infection alters the levels of the low density lipoprotein-related receptor 1 (LRP1), a plasma membrane receptor that regulates lipid and cholesterol metabolism [58].

HCMV-induced reprogramming of lipid metabolism is essential for the success of viral infection. Inhibition of the fatty acid biosynthetic enzymes ACC [18,53] and FAS [18], greatly impairs HCMV replication most likely via the attenuation of virion envelopment [18,53]. Furthermore, the inhibition of multiple FA metabolic enzymes involved in the production of VLCFAs impairs viral replication [56]. Additionally, the inhibition of ELOVLs, specifically ELOVL7, impairs the production of viral progeny [55,56] and the infectivity of the generated virions [55,56]. Interestingly, the detrimental effects of ELOVL inhibition can be rescued with either VLCFA supplementation [55,56] or ELOVL7 overexpression [55], suggesting that HCMV-induced FA elongation is essential for infection and highly dependent on the induction of ELOVLs. In addition, inhibition of the cleavage/activation of the lipogenic transcription factor SREBP1 reduces viral growth [54]. Notably, FA synthesis, although important for early growth and development, is not acutely essential in mammals [59,60,61] and, as such, highlights this pathway as a potential target for therapy development. Sterol metabolism also seems to be important for HCMV infection, as depleting cholesterol from virions reduced their infectivity by blocking fusion of the virion envelope with the cell membrane [58]. In addition, it was reported that siRNA or antibody-mediated inhibition of LRP1 generated virions that contained elevated cholesterol, resulting in enhanced infectivity [58].

The mechanisms responsible for HCMV-induced activation of lipid metabolism are not completely understood. It is clear that HCMV infection requires ACC1 and ELOVLs (specifically ELOVL7) [53,54,55] to induce FA biosynthesis and elongation. It is likely that HCMV induces the expression of lipogenic enzymes through the activation of SREBPs. HCMV infection was shown to induce the maturation and activation of SREBP1 [54,55] and SREBP2 [53], which regulate many SRE-containing lipogenic genes, such as LRP1 [62], ACC and ELOVLs [50,51]. Previous work has shown that SREBP inhibition reduces lipid synthesis and ELOVL7 expression, with SREBP1 inhibition having the greatest effect [54,55]. The upstream mechanism by which HCMV activates SREBPs and their downstream lipogenic effectors is still not well understood, although the activation of the ER stress kinase, PERK, appears to be involved [63]. Previous work determined that gene expression but not DNA replication is necessary for the induction of ACC1 observed during infection [53]. Further, the viral immediate early protein U_L_38 was implicated as a regulator in HCMV-induced lipid reprogramming. Infection with a mutant HCMV virus lacking the U_L_38 gene fails to induce FA elongation and ELOVL7 expression to the levels observed for wild-type infection [55]. U_L_38 overexpression partially induces ELOVL7 expression levels, but its expression is not sufficient to increase FA elongation, suggesting that UL38 is not the sole mechanism by which HCMV induces FA elongation and ELOVL7 expression [55]. As mentioned previously, the U_L_38 protein was reported to bind and antagonize TSC2, thus allowing mTOR activation even under inhibitory conditions [21,22]. mTORC1 is required to maintain active translation and was implicated in the control of key aspects of lipid metabolism via several processes, including the activation of SREBPs [25,26,27,64,65]. Previous work has demonstrated that HCMV infection activates mTORC1 and that maintenance of this activity is required for high-titer viral replication [23,24]. Interestingly, the pharmacological inhibition of mTOR completely abrogates HCMV-induced FA biosynthesis and ACC1 expression, as well as SREBP2 activation [53]. mTOR inhibition also reduced HCMV-induced FA elongation, VCLFA production and ELOVL7 upregulation [55]. Thus, HCMV could be inducing FA biosynthesis and elongation by the activation of the mTOR pathway, and subsequent stimulation of SREBPs, to ultimately turn on SRE-containing genes such as ACC1 or ELOVL7.

Although the mechanisms are still being elucidated, it is clear that HCMV reprograms cellular lipid metabolism and that it is essential for productive infection. The induction of FA biosynthesis seems to be a requirement for late stages of HCMV replication, i.e., after DNA replication [18]. Several late processes during infection might require extra FAs. First, FA biosynthesis might be necessary for the bulk production of new FAs required for viral envelope phospholipids [66]. In addition, newly synthetized FAs could be required for the formation of the viral assembly complex, a highly vacuolated region adjacent to the nucleus that is essential for virion assembly and egress [67]. Additionally, de novo FA biosynthesis might be a requirement for lipid-based post-translational modifications of viral proteins and cellular cofactors, important for productive infection [66]. Lipid modifications, such as myristoylation, were described to be important for the localization and function of HCMV proteins such as pp28 [44]. HCMV-induced reprogramming of lipid metabolism is also essential for the construction of proper functioning virions. Previous work suggested that depleting cholesterol from virions reduced their infectivity by blocking fusion of the virion envelope with the cell membrane [58]. In addition, previous studies have shown that HCMV virions are enriched in saturated VLCFAs [56]. In this regard, cholesterol and VLCFAs could be involved in particle assembly or viral entry into host cell in subsequent replication cycles [66], which is supported by the observation that elongase inhibition results in viral particles with reduced infectivity [56]. In line with this, studies have shown that certain lipids can favor membrane curvature or other biophysical features [68], which might be necessary for virion assembly and budding [66]. Cholesterol and VLCFAs could contribute to the organization of lipid-embedded virion envelope proteins or to membrane dynamics during entry or egress of the virions [66]. All in all, it appears likely that HCMV infection requires the induction of lipid metabolism to not only satisfy the demand for specific lipids required for the generation of new viral envelopes, but also for proper virion assembly, budding or entry into uninfected cells.

## 6. HCMV Infection Induces Prostaglandin Metabolism

**Figure 4:**
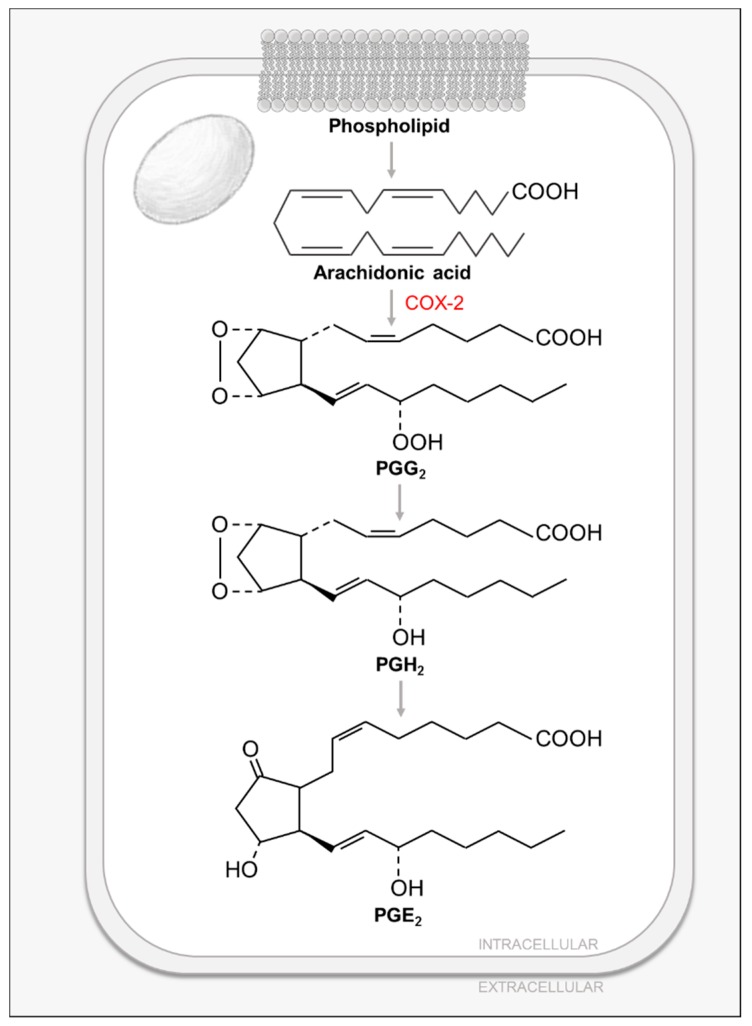
Prostaglandin Biosynthesis.

Lipids play critical roles in mammalian cells, not only as energy storage and scaffolding molecules, but also as precursors to secondary metabolites and messengers. Prostaglandins (PG) are an important class of FA-derived signal-transducing small molecules. Almost all mammalian cells except red blood cells produce PGs and their related compounds, eicosanoids: prostacyclins, thromboxanes, leukotrienes and lipoxins [11]. These compounds have several essential functions such as mediating inflammatory responses, producing pain and fever, regulating blood pressure, and inducing blood clotting [11]. In humans, the most prevalent PG precursor is arachidonic acid (AA). AA is a C_20_ polyunsaturated FA that is stored in cell membranes, esterified to glycerol in phospholipids and is released by the activity of phospholipases, such as cytosolic phospholipase A2 (cPLA2) [11] (Figure 4). cPLA2 is regulated by several processes: first, an increase in intracellular calcium triggers its membrane binding, followed by its phosphorylation by mitogen-activated protein kinases (MAPKs), further contributing to its activation [69]. Released AA is then converted to PGs, prostacyclins or thromboxanes by the prostaglandin endoperoxide (PGH) synthase (PGHS) in the so-called cyclic pathway of eicosanoid metabolism [11]. This multi-step pathway is catalyzed by the two different enzymatic activities of PGHS: a rate-limiting cyclooxygenase activity which catalyzes steps 1 and 3 to generate prostaglandin G_2_ (PGG_2_); and a peroxidase activity which catalyzes the last step and produces prostaglandin H_2_ (PGH_2_), which is then transformed to either prostaglandin E_2_ (PGE_2_) or PGD_2_ (via prostaglandin isomerases), prostacyclin (via prostacyclin synthase) or thromboxane (via thromboxane synthase) [11] (Figure 4). The cyclooxygenase activity of PGHS gives it its common name, COX [11]. PGHS has two isoforms, COX-1 and COX-2, which share 60% of sequence identity and structural homology [11]. COX-1 is constitutively expressed in most mammalian tissues and allows for the basal PG synthesis required to maintain organ and tissue homeostasis [11]. On the other hand, COX-2 is only expressed in certain tissues in response to inflammatory signals such as cytokines, protein growth factors and endotoxins [11]. Non-steroidal anti-inflammatory drugs (NSAIDs), such as aspirin or ibuprofen, inhibit the cyclooxygenase activity of PGHS by blocking its active site channel, thus inhibiting the production of PGs, prostacyclins and thromboxanes [11]. In contrast to NSAIDs that target both COX isoforms, more specific inhibitors known as coxibs, specifically target the inducible COX-2 isoform and its pro-inflammatory effects [11].

The first study to examine the effects of HCMV infection on PG metabolism demonstrated that HCMV infection induces the secretion of AA [70,71,72] and this induction was inhibited by the use of cPLA_2_ inhibitors [72], whose phosphorylation and activity were found to be induced during infection [71]. In line with the previous statement, it was shown that HCMV infection induces mRNA, protein and activity of cellular COX-2 [73], as well as increasing the levels of its product PGE_2_ [70,73]. Interestingly, the observed induction on PGE_2_ levels was sensitive to the use of COX-2 inhibitors [73].

HCMV-mediated induction of PG metabolism is required for successful replication. Initial studies demonstrated that the COX inhibitors, aspirin and indomethacin, negatively impact HCMV infection by inhibiting IE gene expression and viral replication [74]. Later studies have shown that COX-2 inhibition greatly reduces virus production and viral DNA replication, and impacts viral protein accumulation [73]. Interestingly, this inhibition can be rescued by the addition of the COX-2 product PGE_2_ [73]. These data suggest that productive HCMV infection requires increased COX-2 activity and PGE_2_ levels [73]. Further supporting the importance of COX-2 activity for CMV infection, rhesus CMV (RhCMV) encodes a viral COX-2 homolog that was shown to be a critical determinant for the endothelial cell tropism of RhCMV [75].

Although important for successful infection, the mechanisms HCMV employs to manipulate PG metabolism are still unclear. Previous work described that HCMV-induced AA release required viral protein synthesis and occurred under conditions consistent with the expression of HCMV IE genes [70], but the viral factors involved in this induction are still not known. Some studies have linked AA release with tumor necrosis factor alpha (TNF-α) production, as both antibodies against TNF-α and pentoxyphylline (an inhibitor of TNF-α synthesis) inhibited AA and PGE_2_ release [70]. In addition, HCMV-induced MAPK activation was linked to cPLA_2_ activation during infection [71]. Given these findings, a possible mechanism through which HCMV could be inducing PG metabolism is by increasing cPLA_2_ expression via nuclear factor-κB (NFκB)/TNF-α-mediated MAPK activation [76]. In addition, the HCMV UL37x1 protein could be inducing cPLA_2_ activity, given that UL37x1 mobilizes Ca^2+^ from endoplasmic reticulum stores [77], which would be predicted to activate cPLA_2_.

## 7. Conclusions and Future Directions

Over the past several years, it has become evident that HCMV induces many changes to the host cell metabolic network. Further, the interactions between HCMV and the small molecule metabolic network are absolutely essential for successful infection. These viral interactions with the metabolome are diverse and occur over numerous different metabolic pathways, contributing to infection in various ways. In many cases, with the exception of U_L_38, very little is known about the viral factors that are responsible for these metabolic modulations. As the responsible viral factors emerge, they will surely provide information not only towards our understanding of HCMV-mediated metabolic modulation, but also towards our basic understanding of cellular metabolic regulation.

In addition to the viral mechanisms responsible for metabolic remodeling, many questions remain about the precise contributions that specific metabolic activities make towards infection. While some of these links are apparent, e.g., nucleotide sugar metabolism contributing to envelope protein glycosylation, in many cases how these metabolic changes contribute to viral infection are less clear. With the exception of prostaglandin metabolism, we have largely limited our speculation about these metabolic contributions to providing the energetic and biomass requirements of infection. While elucidating these energetic and biomolecular contributions is important, other equally important metabolic contributions to infection likely exist. There was a rapid expansion in the appreciation of how small molecule metabolism contributes to numerous facets of cell biology. These include the metabolic control of signal transduction and gene expression (reviewed in [78]), control of innate and adaptive immunity (reviewed in [79,80]) and the control of stem cell fate and differentiation (reviewed [81]) among others. Steps to elucidate these metabolic contributions will expand our understanding of important areas in viral and cellular biology, in addition to identifying potential therapeutic vulnerabilities to limit morbidity associated with HCMV infection.

## Abbreviations

GLUT4—glucose transporter type 4; FBP—fructose 1, 6-bisphosphate; DHAP—dihydroxyacetone phosphate; 3PG—3-phosphogylcerate; PEP—phosphoenolpyruvate; PFK—phosphofructokinase; PDH—pyruvate dehydrogenase; PK—pyruvate kinase; AMPK—AMP-activated protein kinase; CaMKK—Ca2+/calmodulin-dependent protein kinase kinase; TSC2—tuberous sclerosis protein complex 2; mTOR—mammalian target of rapamycin; mTORC1— mammalian target of rapamycin complex 1; mtDNA—mitochondrial DNA; MRM3—mitochondrial RRNA methyltransferase 3; GLS—glutaminase; GDH—glutamate dehydrogenase; CDP-DAG—CDP-diacylglycerol; UDP-GlcNAc—UDP-N-acetyl-glucosamine; CAD—carbamoyl phosphate synthetase-aspartate transcarbamylase-dihydroorotase; DHO—dihydroorotate; DHODH—Dihydroorotate dehydrogenase; PRPP—phosphoribosyl pyrophosphate; DAG—diacylglycerol; PIP3—phosphatidylinositol-3,4,5-trisphosphate; OAA—oxaloacetate; ACL—ATP-citrate lyase; ACC—acetyl-CoA carboxylase; FAS—fatty acid synthase; LCFA—long chain fatty acid; VLCFA—very long chain fatty acid; HMG-CoA—3-Hydroxy-3-methylglutaryl coenzyme A; SREBP—Sterol regulatory element binding protein; SCAP—SREBP cleavage activation protein; LRP1—lipoprotein-related receptor 1; cPLA2—cytosolic phospholipase A2; MAPK—mitogen-activated protein kinases; PGHS—prostaglandin endoperoxide synthase; NSAID—Nonsteroidal anti-inflammatory drug; TNF-α— Tumor necrosis factor alpha; NFκB—nuclear factor-κB.
